# Clinical classification in mental health at the cross-roads: which direction next?

**DOI:** 10.1186/1741-7015-11-125

**Published:** 2013-05-14

**Authors:** Ian B Hickie, Jan Scott, Daniel F Hermens, Elizabeth M Scott, Sharon L Naismith, Adam J Guastella, Nick Glozier, Patrick D McGorry

**Affiliations:** 1 Clinical Research Unit, Brain & Mind Research Institute, University of Sydney, 100 Mallett Street, Camperdown, 2050, Australia; 2 Academic Psychiatry, Institute of Neuroscience, Newcastle University, Newcastle upon Tyne, NE1 7RU, UK; 3 FondaMental Foundation, Fondation de Coopération Scientifique Hôpital A. Chenevier, 40 Rue de Mesly, Creteil, F-94000, France; 4 INSERM, U 955, IMRB, Psychiatry Genetic, Creteil, F-94000, France; 5 School of Medicine, The University of Notre Dame, 160 Oxford Street, Darlinghurst, Sydney, 2010, Australia; 6 Centre for Youth Mental Health, University of Melbourne, 35 Poplar Road, Parkville, 3052, Australia; 7 Orygen Youth Health Research Centre, Department of Psychiatry, University of Melbourne, 35 Poplar Road, Parkville, 3052, Australia

**Keywords:** Classification, Clinical staging, Mental health

## Abstract

**Background:**

After 30 years of consensus-derived diagnostic categories in mental health, it is time to head in new directions. Those categories placed great emphasis on enhanced reliability and the capacity to identify them via standardized checklists. Although this enhanced epidemiology and health services planning, it failed to link broad diagnostic groupings to underlying pathophysiology or specific treatment response.

**Discussion:**

It is time to adopt new goals that prioritize the validation of clinical entities and foster alternative strategies to support those goals. The value of new dimensions (notably clinical staging), that are both clinically relevant and directly related to emerging developmental and neurobiological research, is proposed. A strong emphasis on ‘reverse translation’ (that is, working back from the clinic to the laboratory) underpins these novel approaches. However, it relies on using diagnostic groupings that already have strong evidence of links to specific risk factors or patterns of treatment response.

**Summary:**

The strategies described abandon the historical divides between clinical neurology, psychiatry and psychology and adopt the promotion of pathways to illness models.

## Background

Globally, the biggest challenge in public mental health is implementing strategies to reduce the societal burden of mental disorders [1,2]. For those living with mental illness and their families, the most pressing issues are increasing access to affordable health care, promoting social inclusion, supporting economic productivity and reducing premature mortality [1-4]. From a clinical perspective, our greatest failure has been the lack of development of new or better-targeted treatments, particularly for those with persisting and disabling disorders. Additionally, our inability to predict individual responses to treatments and our failure to intervene early to reduce disability or prevent premature death are notable [5-10].

Despite the degree of public or media attention [11-13], deciding the immediate fate of our major international classification systems is not our most central task. It is timely, however, to reflect on the Diagnostic and Statistical Manual (DSM) for Mental Disorders, fourth edition [14] and the closely related International Classification of Diseases, 10^th^ revision (ICD-10) [15]. The application of these systems has improved the reliability of syndromal (symptom-based) diagnoses as used in psychiatry, but these remain inferior to the evidence-based etiological or pathway models employed for other common medical conditions.

After 30 years of clinical research, which is based on a common set of principles, our diagnostic entities remain poorly validated. That is, despite the research explosion in basic neuroscience, imaging and molecular genetics, our categories do not link strongly to any specific neurobiological or environmental risk factors, underlying pathophysiological processes, or patterns of specific treatment response [5,6,16-18]. Consequently, our classification systems have arrived at a fundamental cross-road.

While the DSM processes seem set to continue down familiar paths [19], it is timely to consider whether clinical psychiatry and psychology would be better served by heading in somewhat different directions [20-26]. Here, we explore specific alternatives including a much greater fusion of classification systems with other areas of clinical neuroscience, the use of dimensional measures of behavioral and cognitive change, the adoption of a limited number of pathophysiologically based syndromes that incorporate objective testing, a greater reliance on implications of response to specific treatments, and the extent to which incorporation of a common general medical concept - namely, clinical staging - represents a major advance [27,28]. Most importantly, we suggest that such alternatives take us a much greater way along the path to enhanced treatment planning.

### The clinical challenge

It is frustrating that diagnoses are based largely on descriptive phenomena. Often they vary from practitioner to practitioner, and may well change markedly over the course of illness (for example, non-psychotic to psychotic syndrome or unipolar to bipolar mood disorder). As diagnoses are made at non-specific time points along complex illness pathways that evolve from risk to onset and progression to chronic ill-health, they often relate poorly to the actual stage of illness. For the major anxiety, mood or psychotic disorders, the illness process typically has its onset in late childhood or early puberty and then recurs or continues progressively into adult life [29-31]. Although 75% of major mental disorders begin before the age of 25 years [32], our diagnostic criteria are derived largely from the experiences reported by middle-aged persons with established illness. These phenotypes often map poorly onto earlier and often less specific phases of the illness experience [22,25,33,34].

The current systems also assume the concept of multiple parallel pathways each leading to distinct diagnostic categories - an assumption that is not readily supported by modern family, genetic and neurobiological risk factor studies [20,35,36]. Criterion-based symptom sets (which then give rise to specific and ‘independent’ categories) prioritize phenomena such as delusions, hallucinations, periods of elevated mood or increased energy, psychomotor slowing, emotional blunting, or cognitive slowing for disorders such as schizophrenia, bipolar disorder or severe depression. Data from recent community studies that assess patients longitudinally from childhood or adolescence [29,37-39], however, emphasize the extent to which many of these phenomena are shared across disorders [40]. Prototypically, anxiety disorders that are evident in children before the age of 12 years predict later depressive, bipolar and psychotic disorders [41].

With regards to adult-type disorders, persistence or recurrence of symptoms appears to have greater predictive significance than cross-sectional observation of specific symptoms [39,42-45]. Hence, the great clinical challenge is to derive new diagnostic systems that are not only consistent with developmental epidemiology and neurobiology but also useful when applied in everyday clinical practice.

### A historical perspective

The great virtue of the research-based classification systems of the late 1970s was that they promoted the pursuit of reliable diagnoses. To achieve diagnostic reliability, a small number of dimensions in human behavior (for example, anxiety, depression, impaired cognition or psychotic phenomena) were organized into a large number of discrete and separate ‘disorders’ - on the basis of the presence or absence of set numbers of key symptoms. Inevitably, this gave rise to a checklist approach to diagnostic practice and the proliferation of diagnostic ‘categories’.

However, reliability was oversold as the necessary precursor to validation of those entities [16]. While the ‘atheoretical’ and reliability-driven approach of DSM-III did set clinical research free from the previous psychological, behavioral or medical models, it did not result in a new era of preventive and therapeutic strategies. In retrospect, it appears that the DSM-III-derived disorders or independent categories relied too heavily on descriptive psychopathology, historical practice or clinical consensus [46,47].

The post-DSM-III era did enhance our capacity to conduct large, multi-site and international research and ensured that diagnostic concepts were less constrained by local history, culture, religion or social customs, or fashion. Such aspirations were essential to promoting the international ‘science’ base of clinical psychiatry and psychology and for forging links to key areas of emerging neuroscience, especially molecular genetics and neuroimaging. Further, this greatly assisted the move away from highly idiosyncratic practice or ‘diagnostic systems’ that were used to prop up the delivery of poorly evaluated (or intrinsically harmful or discriminatory) forms of mental health care. Mental health practice is one area of social endeavor that has benefited greatly from globalization and greater transparency, communication and scrutiny [2].

When linked to national epidemiological surveys, the international classification systems have underpinned more accurate estimates of relative disease burden, access to care and impacts of mental disorders on broader health and social systems [1,32,48]. This has led to more forward thinking about how to best support broad social settings that enhance mental health and wellbeing [49]. Thus, in many ways, the great successes of the post-DSM-III diagnostic era are in public health and related health system developments. It is rather frustrating that, despite the best efforts of scientists and practitioners, the stated aims of advancing clinical research have not been so successful.

## Discussion

### Setting new goals for international classification systems

Given the relative failure to validate the DSM- or ICD-derived categories, it is time to set new goals (Table 1) - and propose a range of possible, and rather diverse, strategies to support those goals (Table 2). An over-riding concern in mental health has become the reduction of the population-health burden by adoption of various early intervention strategies [28,50,51]. These focus clinical service development and related research on identifying early forms of illness not only to reduce current morbidity but also to prevent progression to more severe or chronic illness types and associated role impairment [52-57]. Although the fundamental empirical work in this area has focused largely on psychotic disorders, there is now a much broader clinical database emerging that is targeted at the early phases of the more prevalent anxiety and depressive disorders [22,53,58-61].

**Table 1:** New goals for enhanced diagnostic categories and worked examples for major depression

**Clear goals**	**Consequences for diagnoses related to major depression**
1. Focus primarily on enhancing clinical practice	1. Abandon use of the single term ‘major depression’ as on its own it does not predict response to specific psychological or physical treatments [62];
	2. Only use in association with specifiers that predict likely response to specific treatments - for example, major depression with psychotic features; melancholia associated with psychomotor changes [63-65]; depression following manic episode [62];
	3. Differentiate other risk or comorbidity factors from the diagnosis itself - notably risk of self-harm or suicide or misuse of alcohol and other substances [66-69].
2. Link directly to objective markers of pathophysiological processes	Require the cross-sectional and longitudinal recording of objective markers that may predict response to treatment or risk of recurrence:
	• Neurohormonal - for example, presence of non-suppression to dexamethasone [70];
	• Circadian or sleep - for example, actigraphic evidence of phase-delay [58,60,71];
	• Psychomotor change - for example, observer or automated measures [63-65];
	• Neuropsychological - for example, neuropsychological evidence of delayed reaction time [72-75];
	• Brain imaging - for example, presence of subcortical white matter changes [59,74,76-82].
3. Incorporate known facts about developmental paths, environmental risk factors, course of illness or family history	1. Differentiate early-onset (<30 years) from late-onset (>50 years) forms [83-87];
	2. Differentiate first major episode from recurrence, relapse or chronicity [21,22,52,88];
	3. Record clear environmental (for example, seasonal onset, exposure to traumatic events) or medical illness (for example, post-stroke) exposures that are concurrent with depression [89];
	4. Record clear earlier (notably childhood) phenotypes such as childhood anxiety;
	5. Record clear family history data related to presence of psychosis, mania or suicide in first-degree relatives [35,37,90-95];
	6. Record clear history of exposure to social adversity or interpersonal stressors or ongoing evidence of major socio-economic, interpersonal or other relevant social circumstances [96-98].
4. Be consistent with data from family, twin or genetic studies	1. Restrict the diagnosis to those sub-categories with strong evidence of high heritability - for example, depression in those with previous mania; depression in those with psychotic features [35,37,38,99-101];
	2. Support the concepts of depressive disorders preceded by childhood anxiety or early- versus late-onset depressive disorders [66,84-86,102].
5. Capture key aspects of illness stage	Use a clinical staging format for depressive disorders that differentiates early stages that are strongly linked to other childhood and adolescent phenotypes (for example, anxiety, phase-delay in sleep and circadian systems, fatigue, hypomania, mood instability) from later early adult or mid-adult stages (which may also be associated with different phenotypes such as psychomotor change, phase-advance in sleep and circadian systems) [21,22,58-61,88,102].
6. Best predict future illness course or response to specific treatments.	Use known factors about response/non-response to specific treatments - for example, for acute episode: classify as selective serotonin reuptake inhibitor responder or non-responder; classify as responder or non-responder to cognitive behavioral therapy [103-105].

**Table 2:** New strategies for deriving diagnostic categories and worked examples for major depression

**New strategies**	**Implications for major depression**
1. Abandon the artificial distinction between brain (neurological) and psychiatric or psychological (mental) disorders	1. Focus clinical attention on the broad affective, cognitive, motor and sleep or circadian aspects of significant depressive disorders [58-61,72,106];
	2. Encourage systematic cross-sectional and longitudinal structural brain imaging across the various phases of early- and late-onset depressive disorders [78,83].
2. Avoid the use of single categorical states (for example, major depression, schizophrenia, bipolar disorder) that describe heterogeneous groups	Only use major depression in association with specific descriptors including early- versus late-onset, preceded by childhood anxiety; comorbid with alcohol or other substance misuse, significant circadian disturbance, psychotic features, significant psychomotor disturbance or other discrete melancholic features [61,63-65,78,84,107-110].
3. Promote pathways to illness models that have a strong basis in longitudinal epidemiology and related risk factor or neuroscience research	Promote categories such as depression preceded by childhood anxiety; childhood traumatic events; depression associated with significant circadian disturbance; depression associated with psychomotor change; depression following a clear manic episode [61].
4. Incorporate age-of-onset and stage-of-illness concepts into all diagnostic processes	1. For depression, the first age of a clear depressive syndrome would be recorded, as well as the first clear episode of sufficient severity to justify intervention [66,111,112];
	2. For depression, the clear pattern of remission, relapse or recovery would be recorded for all patients [113].
5. Reduce the concept of comorbidity to the co-occurrence of genuinely independent conditions	1. Depression occurring in association with documented diabetes or cardiovascular disease [114-119];
	2. Rejecting the notion of anxiety and depression representing comorbid conditions, as distinct from linked developmental phenotypes [61].
6. Place greater importance on the significance of response to specific treatments	1. Patients with anxiety and depression who fail to respond in the acute phase to CBT but do respond to an SSRI or SNRI can be considered to be in a different category [103,104,113];
	2. Patients with psychomotor change or cognitive impairment who do not respond to SSRI or SNRI but do respond to physical treatments such as electroconvulsive therapy can be considered as a different category [64,120];
	3. Patients with sleep or circadian disturbance who fail to respond to respond to CBT or SSRI or SNRI but do respond to behavioral or pharmacological management that targets the circadian system can be considered to be in a different category [121-124].

CBT: cognitive behavioral therapy; SNRI: selective norepinephrine reuptake inhibitor; SSRI: selective serotonin reuptake inhibitor.

This movement is similar in conceptualization with that being promoted in other related clinical areas of medicine that have very large impacts on current and future health costs, notably diabetes and related metabolic syndromes [125-127]. As with the move to define pre-diabetes (an intermediate state between normal and clearly raised blood glucose concentrations), it is entirely possible to define emerging mental disorders by their intermediate symptom levels, impacts on function, patterns of persistence over time or predictive capacity [21,22,56,57]. Further, it is possible to design new health care platforms to increase the access to care for young people presenting with these conditions [53,55].

Importantly, the same types of concerns arise in the arenas for both pre-diabetes and early intervention in mental health [51,128]. That is, as the number of individuals identified by health systems grows, and those with less severe forms come into active clinical care, the extent to which early forms of illness can be effectively managed largely by non-pharmacological or other lifestyle-based approaches is a key consideration [66,129,130]. Critics of this area [11,131] all too readily see an over-medicalization or a pharmaceutical-industry-driven conspiracy to be at the heart of such genuine public health movements. Yet the evidence from the clinical staging of ischemic heart disease largely demonstrates the opposite. The greatest public health emphasis was placed on reducing smoking rates, promoting exercise and addressing other modifiable risks. High cost, high risk interventions (for example, revascularization) were reserved for those experiencing major events (for example, first myocardial infarct), those with recurring episodes (for example, coronary by-pass surgery), or following a progression to chronic disease (for example, pharmacotherapy of heart failure) [132,133].

Currently, the key difference between the diabetes and early mental disorder arenas is the extent to which the diagnosis of the ‘at-risk’ or ‘prodromal’ state for diabetes relies on an independent laboratory test - and the extent to which that laboratory test has predictive value for later poor health outcomes. Therefore, concurrently with the development and evaluation of earlier diagnostic thresholds based on descriptive phenomena and related disability estimates [22,52,53], we must intensify our search for better neuropsychological, brain imaging, circadian, neurophysiological, immune or other markers of these early states [58,59,72,76,99,106,134-136] and design studies to test their predictive capacity.

These new goals respond directly to the challenges posed by novel preventive and longitudinal approaches that are targeted at the recognition of early phenotypes. For example, for depressive disorders, there is international recognition that the extraordinary premature death and disability costs attributable to these conditions [48,137] reflect their early age-of-onset, high current and lifetime population prevalence, typical illness patterns of recurrence and chronicity, and likely comorbidity with alcohol and other substance misuse as well as physical ill-health, most notably in the form of premature cardiovascular disease [90,107,138-141]. The ways in which incorporating these new goals might impact on the classification of depressive disorders is outlined here (see Table 1).

As importantly, it is necessary to state those factors which are not the primary goals of a clinical or research classification system. These include: categorizing all forms of abnormal perception, mood, cognition or other behavioral disturbance; imposing independent category status on disturbances that are fundamentally dimensional in nature and frequently inter-correlated (see common forms of anxiety and depression [142,143]); creating diagnostic hierarchies that presume etiological or pathophysiological dominance of one form of disturbance over another (for example, psychotic compared with mood or cognitive phenomena); or using the classification system as the sole basis for allocating research funds, licensing treatments, providing access to health care, determining legal matters, reimbursing health care costs or supporting access to other personal entitlements system.

Contrary to prevailing wisdom, there is no urgent need to have one over-arching international diagnostic system or bible for all perceptual, mood, cognitive and other behavioral syndromes. Unfortunately, the recent attempts to include all things within the one descriptive system have underpinned a rather unhelpful debate about the inclusion or exclusion of fewer or more categories [11,12,144]. Another unintended side-effect of the forced international orthodoxy was the lack of support of other plausible models or active support for the development of alternative approaches [13,16,145].

An emerging body of work, utilizing appropriate genetic modeling and brain imaging data provides provisional support for such alternative approaches [20]. These data favor a model in which mental disorders represent broad patterns of psychopathology. Within this model, various genetic and environmental factors determine the development of common brain circuits that underpin normal behavior and cognition. When perturbed, changes in the function of these circuits give rise to deviant behavior or cognitive function. This model has influenced the National Institutes of Mental Health, which now actively promotes investigation of brain-circuit-based approaches and proposes that they may underpin a new approach to the classification of common mental disorders [20,146].

Within the circuitry models, changes from normal function (thereby giving rise to disorders) are by their nature dimensional rather than categorical. Generally, correlations exist between the degree of accumulated dysfunction of the underlying circuits (or related systems) and the extent of behavioral or cognitive change. This dimensional approach shifts the emphasis from making a specific diagnosis to determining the current level of cognitive or behavioral change in individual patients. Clinical and related neurobiological research then examines the extent to which common risk variables (for example, developmental delay, trait neurocognitive impairments, alcohol or other substance misuse) increase risk to measured behavioral or cognitive change, increase risk to markers of brain impairment (for example, poor neuropsychological function, excessive cortical thinning, disturbed neurophysiological markers) or increase risk to poor outcomes - independently of any specific relationship with classical ‘diagnostic’ entities [108,147,148].

Core dimensions of cognition and behavior are hypothesized to be common to all persons and not viewed as a unique set of characteristics occurring only in those who present with mental health problems. Perturbations of brain circuits will typically result in ‘trans-diagnostic’ rather than pathognomonic symptom sets. An important prediction of such systems is that many objective measures of the structure (for example, brain imaging) or outputs of such systems (for example, cognition - including traditional neuropsychological and social cognition, circadian, hypothalamus-pituitary-adrenal axis function, neurophysiological or immune) will also show little diagnostic specificity. Over the last 30 years, this has been the overwhelming experience with proposed diagnostic markers, such as the dexamethasone suppression test for major depression [149]. Preliminary analyses of data from those in the early phases of a range of major psychotic or mood disorders also support this perspective [58-61,72,73,76,99,106,136,150],[151].

### New strategies for enhancing the diagnoses of perceptual, mood, cognitive and other behavioral syndromes

A range of diverse strategies can be advanced to support these new goals (see Table 2). These do not need to come together into one single bible for clinical or research purposes, as the initial aim is to develop, evaluate and refine them frequently as evidence emerges. A potential consequence of the key shift from many categories to the use of fewer underlying dimensions is that it is likely to lead to the need for an alternative term to mental disorders. A very broad descriptive concept that encapsulates a 21st century approach to disturbances of perception, mood, cognition and other behavioral dimensions may be required. The clear public, professional, clinical and research benefits likely to derive from abandoning the historical and cultural divisions between clinical neurology and psychiatry and clinical psychology have been highlighted by others [13,26].

In doing so, such strategies move away from the very broad categories we currently use (for example, major depression) to describe very heterogeneous populations. Generally, more specific states (for example, depression in association with late-life vascular disease; first episode psychosis; cannabis-associated recurrence of psychosis; depressive episode following previous manic episode) provide a better fit with known risk factors, actual pathophysiology or known illness course and may be much more useful in research and some fields of clinical practice [83].

In association with all disorders, more general age-of-onset and stage-of-illness concepts should be recorded. Those that have clear pathways to illness need to be emphasized (for example, childhood attention deficit hyperactivity disorder leading to early age of onset of substance misuse; childhood schizotypal or autistic behaviors leading to early onset psychotic disorder; adolescent social anxiety leading to alcohol misuse). Concurrently, this may help to reduce the overuse of the concept of comorbidity - limiting it in the future to the co-occurrence of genuinely independent conditions (for example, psychotic disorder and alcohol dependence).

It is clear that one of the most robust ways of differentiating diagnostic groups is on the basis of response (or non-response) to specific treatments. Much greater effort needs to be invested in the reverse translation agenda (that is, working back from the bedside to the bench). For example, we urgently need to unravel what neurobiological factors differentiate those with schizophrenia who respond to clozapine or those with bipolar disorder who respond to lithium. The goal is to develop better predictors of treatment-response before initiating therapies.

At this time, we argue that we may be better served by investing heavily in a much smaller number of more discrete syndromes that are partially validated by clinical course, objective markers or predictors of treatment response. That is, rather than prioritize reliability, we could vigorously pursue those more homogeneous groups that are identified in clinical practice or family studies [90-93]. Inevitably, this would advance the push for more personalized approaches to health care. Further, relevant groups could be preferentially recruited to specific preventive or treatment trials (for example, late-onset depression; childhood-onset obsessive-compulsive disorder; psychotic disorder preceded by childhood schizotypal behaviors; first-episode mania; depressive disorders characterized by disturbed hypothalamic-pituitary function, circadian disruption or immune activation).

Another clear way of approaching this desire for greater specificity is to focus on syndromes that are closely linked with discrete environmental exposures, social adversity or inter-current medical events [152]. These natural experiments can be used to facilitate more targeted pathophysiological studies (for example, post-infective or post-stroke neuropsychiatric syndromes; post-traumatic depressive states; depression or prolonged fatigue states following cancer therapies; alcohol or other substance misuse neuropsychiatric syndromes; cannabis-associated first episode of psychosis [89,153-156]).

We would also promote an overt move away from checklist-driven criteria that rely on poorly validated symptom sets (for example, treating independent symptoms such as sleep disturbance and suicidal ideation as equivalent items for the diagnosis of major depressive disorders). Instead, we favor a move back towards clear syndromes that share key pathophysiological, symptom or illness course features (for example, psychotic depression; melancholic disorders that are associated with concurrent psychomotor change; first-episode psychosis; bipolar disorder diagnosed by a discrete manic episode). This process would also be assisted by greater use of objective markers of key features of disorders (for example, computer-generated measures of neurocognitive function; actigraphy-generated measures of sleep-wake cycle; self-report based measures of cognitive styles) and wider use of clinician or self-reported measures of key symptom dimensions.

### Promoting the use of clinical staging

One of the most important developments in recent years has been the importing of the concept of clinical staging from general medicine. In other clinical domains (for example, oncology, coronary heart disease, inflammatory join disease), it is totally inadequate to choose treatments, or plan health care, for persons who suffer from recurring or progressive conditions simply on the basis of a broad diagnostic category (for example, breast cancer). We suggest that it is equally meaningless in mental health to select specific treatments on the basis of broad categories such as schizophrenia, bipolar disorder or major depression. There is a wealth of evidence indicating that patients at different points along the illness continuum of all of these conditions show quite different patterns of response to various interventions [21,88,157,158].

**Figure 1:**
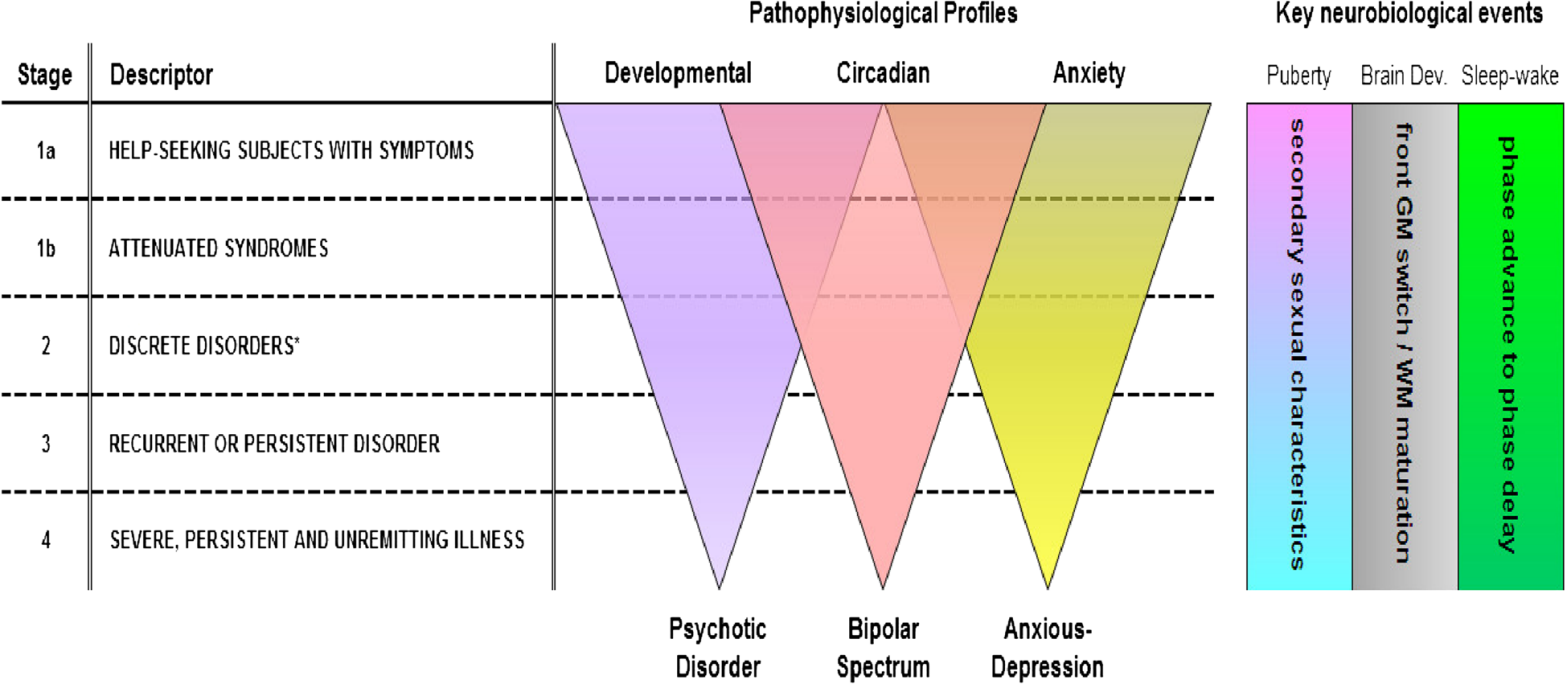
**A clinical staging model for post-pubertal onset and course of major mental disorders: developmental, circadian or anxiety pathophysiological pathways progress from non-specific to discrete syndromes.** *Not necessarily a Diagnostic and Statistical Manual for Mental Disorders, fourth edition, or International Classification of Diseases - 10^th^ revision diagnosis; GM: gray matter; WM: white matter.

Consequently, we have proposed a general framework for clinical staging that can be applied to the more severe mood or psychotic disorders (Figure 1). This framework is readily applied to those who present for health care and clearly differentiates those in early phases (stages 1a ‘seeking help’ or 1b ‘attenuated syndromes’) from those who have reached a higher threshold for disorder (stage 2 and above - see Figure 1). Current clinical and related neurobiological studies (for example, magnetic resonance imaging; neuropsychological and sleep/circadian studies) of this framework provide provisional evidence in support of its reliability and validity [22,58,59].

As compared with current DSM and ICD disorder thresholds, and particularly for the common anxiety and depressive disorders, this approach raises the bar for the initiation of more specific or intensive pharmacological or behavioral strategies. That is, although this approach does encourage more active health care for those at lower levels of illness, it also promotes the use of safe, easily-delivered and non-specific psychological intervention, health-care, suicide prevention and other secondary prevention strategies for those who have not yet reached the higher threshold for a stage 2 disorder [54,84].

### Likely benefits of developing multiple diagnostic dimensions and linking these to clinical staging

All of these strategies move the emphasis from the rather sterile academic debates about the virtues of competing symptom sets (or illness thresholds) [159-161] to the provision of relevant treatment at key points along an illness path. In dialogue with individual patients, this would result in the provision of far more accurate, multi-dimensional and context-relevant information. In our view, stating clearly that a 21-year-old man has a first-onset psychosis, with a strong family history of mania and associated five-year history of persistent cannabis use is more likely to guide treatment selection, health care planning and accurate prognostic statements than assigning any of the current psychosis disorder categories. Similarly, stating that a 17-year-old girl has the recent onset of a depressive disorder characterized by psychomotor slowing and preceded by prolonged fatigue, seasonal changes in mood and energy, with no evidence of childhood anxiety or concurrent alcohol or substance misuse is more useful than attaching any of the current depression categories. While these approaches are already common in everyday clinical practice they are not captured in our current diagnostic systems.

Further, we need systems that support innovative research paradigms such as those promoted by the youth mental health for common mental disorders and early intervention in psychosis movements [162,163]. Within these novel paradigms, it is far more important to recruit patients who share key demographic, illness stage, prior treatment characteristics or family history (for example, age, gender, duration of illness, lack of prior exposure to medical treatments, family history of psychosis or mania) rather than pre-selecting on the basis of poorly-validated syndromal constructs such as major depression.

Such novel research paradigms inevitably focus attention on those genetic or environmental risk factors that are common across disorders (and hence may be modified with resulting benefits to a large number of persons), distinct from the narrow search for unique risk factors that link specifically to each separate disorder. Key considerations such as the role of intra-uterine environments, early childhood infections, childhood sexual or emotional abuse, early adolescent alcohol or substance misuse, grossly distorted adolescent sleep patterns, and physical inactivity in the teenage years will then emerge as major foci for targeted research and potential public health programs [58,60,108,164-166].

### Implications for clinical research and reverse translation

These alternative approaches to classification would give rise to a new wave of early intervention, biomarker, clinical intervention and other longitudinal studies. Specifically, they would also promote reverse translation initiatives - meaning those research programs that work back from clinical research based on well-characterized, more narrow and probably more homogeneous patient groups to elucidate more fundamental biological correlates. Additionally, the reverse translation agenda has other important attributes, including actively responding to topics of real significance to those living with the illness (for example, cognitive impairment in those with psychotic disorders) and capitalizing on phenomena that have been well-replicated in studies on humans (for example, lithium-responsive bipolar disorder or clozapine-responsive schizophrenia).

The combination of key clinical insights and patient priorities plays a crucial role in setting the reverse translation research agenda [2]. Categories deserving of detailed neurobiological research are those clinical situations in which a robust link with relevant pathophysiological, risk, illness-stage or treatment variables has already been demonstrated (for example, circadian-based depressive disorders - see Table 3; cannabis-associated psychosis; first-episode mania; or clozapine-responsive schizophrenia disorders).

**Table 3:** Reverse translation research agenda for circadian-based mood disorders

**Reverse translation strategies**	**Specific research studies**
1. Identify cohorts with clear indicators of circadian-based pathophysiology [58,60,99,167,168]	1. Establish relevant clinical cohorts. For example:
	a. early-onset depression with family history of mania;
	b. less than 10 years of active illness with lithium-responsive mania;
	c. early-onset depression with evidence seasonal change in disorder severity or preferential response to behavioral or pharmacological circadian-based interventions;
	2. Introduce circadian-based phenotypes or markers to other relevant epidemiological, clinical or longitudinal studies. For example:
	a. sleep-wake cycle and circadian phenotypes into relevant developmental, family, genetic or twin studies;
	b. sleep-wake cycle and circadian phenotypes into relevant population-based studies of illness-onset or course.
2. Introduce specific biomarker strategies to the study of cohorts with circadian-based pathophysiology	Introduce objective markers of 24 sleep-wake and circadian activity cycle to descriptive, longitudinal and interventional studies. For example:
	a. use of smart-phone technologies to track sleep cycles;
	b. use of ecological monitoring application technologies to study behavioral rhythm patterns;
	c. use of actigraphy to study timing and stability of activity cycles;
	d. use of dim-light melatonin assays to study patterns of melatonin onset.
3. Design prevention or clinical intervention studies that are relevant to cohorts characterized by circadian-based dysfunction (for example [104,123,125,169-175])	Trial designs include:
	a. selection of young patients with depressive disorders and concurrent phase-delay syndromes for evaluation of efficacy of circadian-based behavioral interventions, light therapy, melatonin or agomelatine, remelton or tasimelteon;
	b. selection of young patients with depression and phase-delay, and family history of mania or psychosis or family history of response to lithium, for evaluation of behavioral or pharmacological strategies to prevent first episode of mania;
	c. evaluating whether those with bipolar disorder who are most responsive to lithium also have depressive disorders that are preferentially responsive to light therapy, melatonin or agomelatine;
	d. evaluating the effects of circadian-based interventions on the course of metabolic parameters in young persons treated for depression or bipolar disorder.
4. Initiate specific genetic or pathophysiological studies in those with specific circadian-parameters [176,177]	Examples include:
	a. specific genetic association for circadian markers in those cohorts who are responsive to lithium or other circadian-specific interventions;
	b. specific genetic association for circadian markers in family members of those with lithium-responsive bipolar disorder;
	c. specific genetic association for circadian markers in family members of those with depression who are responsive to light therapy, melatonin or agomelatine;
	d. evaluate the capacity of cross-sectional and longitudinal dim-light melatonin onset assays to predict the response to treatment or rate of recurrence in those with circadian-based depressive disorders;
	e. evaluate the capacity of cross-sectional and longitudinal dim-light melatonin onset assays to predict the response to treatment or rate of recurrence in those with bipolar disorders.
5. Designing relevant animal model systems to evaluate the likely therapeutic effect of novel behavioral or pharmacological interventions or better understand the effects of effective interventions on the circadian clock [122,178-180]	1. Development of Zebra fish based assays of effects of differing pharmacologies on circadian-dependent locomotor function in fish larvae;
	2. Design studies using effective circadian therapies for mood disorders (as defined by human response) in genetically-informative mice to study changes in underlying mechanisms of the circadian clock and its output systems;
	3. Test novel pharmacological strategies (that is, agents which target molecular mechanisms of the circadian clock) in animal models of depression.
6. Development of novel biomarkers of the circadian system for use in risk factor and treatment systems (for example, [181-185])	1. Optimization of measurements of circadian disruption in humans with major affective disorders, via new systems and technologies (for example, circadian phase in fibroblasts) - with a focus on easy repeatable measures not only of phase-shifts but also internal desynchrony;
	2. Relating measures of disruption of the circadian systems to other measures of chronic distress (for example, hair cortisol measures).

On the basis of making new links in these smaller but clinically-defined cohorts with the best available markers of the active pathophysiology, we would then expect new insights that could provide a basis for working forwards again (that is, bench back to bedside). A new wave of more relevant animal models, molecular targeting or other rapid assay modalities could emerge. The goal then is to use that new knowledge to implement better targeted and more individualized prevention or active treatment strategies (that is, traditional forward translation programs [167,186-188]). Such translational research programs (that is, those that incorporate both reverse and forward strategies) would then genuinely link 21st century neurobiology to clinical practice in an iterative and mutually-informative discourse.

The impact of the move away from investigating traditional schizophrenia to more focused first-episode psychosis (and then related prodromal or at-risk clinical research) in Australia, Europe and the United Kingdom demonstrates the extent to which these novel approaches can genuinely transform clinical practice [27,56,163]. By contrast, those working in the anxiety and mood disorders fields have been slow to comprehend the significance of these developments [28,85]. We believe the time is now right for a more general shift in direction in favor of those diagnostic practices that focus attention on key developmental, illness course, reverse translation and strategic intervention approaches. Although we can recognize the genuine progress that the DSM and ICD revisions from 1980 onwards supported, there is now no longer any good reason for the international mental health community to be constrained by the ongoing revisions of these systems.

## Summary

After 30 years of consensus-based diagnostic categories in mental health, there is great clinical and public frustration with our relative failure to deliver real clinical advances. The approach introduced by the DSM-III (1980) prioritized reliability and imposed a categorical approach on many underlying dimensions of abnormal cognition, mood or behavior. It also sought to ‘cover the field’ and in doing so gave rise to new entities for further research and evaluation. The general approach enhanced epidemiology, international collaboration and health services planning, but too little progress has been made with linking the multiple broad entities to clear genetic or environmental risk factors, underlying pathophysiology or specific patterns of treatment response. Consequently, we have argued that it is time to prioritize the validation of more robust clinical entities and foster the development of specific strategies to support this priority. The proposed goals put enhanced clinical practice back at the center of the endeavor, work with strong cross-sectional and longitudinal epidemiological, family and twin studies, and use available objective markers. The value of implementing new dimensions, notably clinical staging, that are both clinically relevant and directly related to emerging epidemiological, developmental and neurobiological research is also proposed. The strategies described abandon the historical and cultural divides between clinical neurology, psychiatry and psychology and actively move away from broad descriptive terms (for example, major depression, schizophrenia, bipolar disorder) to the promotion of pathways to illness models. These models incorporate age-of-onset, known environmental risk factors and patterns of response to specific treatments (for example, lithium-responsive bipolar disorder, clozapine-responsive psychosis). A strong emphasis on a reverse translation agenda (that is, working back from the clinic to the laboratory) is highlighted. This agenda relies on using diagnostic groupings that already have strong evidence of links to specific risk factors or patterns of treatment response. We have detailed the ways in which these approaches could enhance clinical practice in the most impactful of the major mental disorders, namely major depression. In this worked example, the term major depression would largely be replaced by much more specific terms that differentiate early- from late-onset, those presentations that fundamentally followed-on from childhood anxiety and those disorders that were strongly linked with objective measures of circadian disturbance. As with all other disorders, clinical staging of depressive disorders would also clearly differentiate early-attenuated forms from first major episodes of illness (both typically occurring in adolescence or early adult years) and later recurrent, persistent or chronic forms.

## Abbreviations

DSM: Diagnostic and Statistical Manual of Mental Disorders; ICD: International Classification of Diseases.

## Competing interests

IBH was a director of headspace: the national youth mental health foundation until January 2012. He is the executive director of the Brain and Mind Research Institute (BMRI), which operates two early-intervention youth services under contract to headspace. He is a member of the new Australian National Mental Health commission and was previously the CEO of beyondblue: the national depression initiative. He has led a range of community-based and pharmaceutical industry-supported depression awareness and education and training programs. He has led depression and other mental health research projects that have been supported by a variety of pharmaceutical partners. Current investigator-initiated studies are supported by Servier and Pfizer. He has received honoraria for his contributions to professional educational seminars related to depression, youth mental health and circadian-rhythms research. DFH has received honoraria for educational seminars from Janssen-Cilag. EMS is the (unpaid) Clinical Director of Headspace Services at the BMRI, the (unpaid) Coordinator of the Youth Mental Health Research Program at the BMRI, and Deputy Director of St Vincent’s Private Hospital Young Adult Mental Health Unit. She has received honoraria for educational seminars related to the clinical management of depressive disorders supported by Servier and Eli Lilly pharmaceuticals. She has participated in a national advisory board for the antidepressant compound Pristiq, manufactured by Pfizer. PDM is director of headspace and also of Headstrong (Irish National YMH Foundation) and he holds Investigator Initiated Research Grant Support from Janssen Cilag, Eli Lilly, BMS and Astra Zeneca. PDM also has consultancy and advisory roles with Janssen Cilag, Eli Lilly, BMS, AZ Roche and Pfizer.

## Authors’ contributions

All authors discussed the evidence and contributed to the writing of this manuscript, primarily through a significant discourse undertaken over the past five years. All authors read and approved the final manuscript.

## Pre-publication history

The pre-publication history for this paper can be accessed here:

http://www.biomedcentral.com/1741-7015/11/125/prepub
